# Temperature-Controlled Surface Plasmon Polariton Propagation in InSb–Uniaxial Chiral–InSb Waveguides for Near-Infrared Optical Communication

**DOI:** 10.3390/mi17091099

**Published:** 2026-09-18

**Authors:** Bader Alhasson

**Affiliations:** Department of Electrical Engineering, College of Engineering in Al-Kharj, Prince Sattam bin Abdulaziz University, Al Kharj 11492, Saudi Arabia; b.alhassoun@psau.edu.sa

**Keywords:** surface plasmon polaritons, uniaxial chiral medium, temperature-sensitive material, InSb, near-infrared optical communication

## Abstract

Highly integrated photonic devices have attracted considerable attention for data-transmission systems. However, conventional metal-based photonic devices provide limited tunability and control over electromagnetic surface waves. Therefore, dynamically tunable integrated photonic devices are needed. This paper presents a theoretical model of an indium antimonide–uniaxial chiral–indium antimonide (InSb-UAC-InSb) structure operating in the near-infrared frequency regime. Electromagnetic wave theory is used for numerical analysis, and the characteristic equation is obtained by applying the appropriate boundary conditions. The propagation constant is examined for different values of chirality, core-width, InSb temperature, and incident-wave frequency for two types of uniaxial chiral media. Variation in temperature and chirality demonstrate the tunability of the interface under different operating conditions, enabling enhanced light confinement and low-loss propagation modes in the near-infrared region. The results show that the propagation constant in Case II exhibits greater sensitivity than that in Case I and shows a high value even at lower frequencies. This study provides a promising platform for thermally reconfigurable photonic components, temperature-sensitive optical devices, and near-infrared optical communication applications.

## 1. Introduction

With the rapid advancement of optical technologies, the demand for highly integrated photonic devices for data-transmission systems has increased. In conventional photonic devices, reducing component dimensions to the nanometer scale is difficult because of Abbe’s diffraction limit, which is approximately one half of the optical wavelength. This limitation can be overcome by exciting surface plasmon polaritons (SPPs), which provide a promising approach for manipulating and controlling the dispersion and propagation of light at the nanometer scale [1]. An SPP is an electromagnetic surface wave that travels along a metal–dielectric interface, enabling optical-signal transmission through nanoscale devices beyond the diffraction limit. SPPs have important applications in biomedical sensors, nonlinear nanoscale photonics [2,3], and photolithography [4]. Their characteristics, including wave localization, wavelength, and attenuation along the propagation direction, strongly depend on the material combined with the metal [5]. Various plasmonic nanostructures have been investigated, including isotropic chiral-metal interfaces, where the propagation of hybrid SPP waves has been studied [6]. Yaqoob et al. theoretically investigated the propagation and modulation of hybrid SPPs along a chiral–graphene–metal interface [7]. Zhang et al. studied the propagation, dispersion, and transverse spin of SPPs in a metal–chiral–metal waveguide [8]. Zhang et al. also analyzed the characteristics of SPPs in a dielectric chiral–metal–chiral waveguiding structure [9]. Zhao et al. investigated the behavior of propagating SPPs in a chiral–graphene–chiral waveguide geometry [10]. Although these studies demonstrated the influence of chirality on SPP propagation, most were limited to metal- or graphene-based structures. As a result, dynamic control of SPP characteristics through external physical parameters remains restricted. Chirality refers to the absence of mirror symmetry. An isotropic chiral medium is rotationally symmetric but cannot be superimposed on its mirror image by translation or rotation [11]. Chiral media exhibit exceptional characteristics, such as circular dichroism, circular birefringence, and even negative refraction [12]. A nonchiral object is referred to as achiral. Waveguides based on chiral SPPs have gained considerable attention in the scientific community. However, a major disadvantage of isotropic chiral media is that their degree of chirality cannot be significantly controlled [13,14]. To overcome this limitation and enhance SPP modulation and sensitivity, uniaxial chiral structures have been introduced. A uniaxial chiral medium can be fabricated by incorporating small chiral objects into an anisotropic medium. A uniaxial chiral (UAC) medium is a special type of chiral medium in which chirality appears primarily along one direction [15]. UAC media have different chirality and refractive indices along the perpendicular and longitudinal axes [16]. In such media, material chirality is coupled with intrinsic anisotropy; therefore, the structure lacks mirror symmetry and interacts differently with left- and right-circularly polarized light. In UAC media, chirality-induced coupling between electric and magnetic fields produces hybrid modes that combine the properties of transverse magnetic (TM) and transverse electric (TE) waves. This electromagnetic-field coupling leads to low propagation loss and strong field confinement, which are difficult to achieve in isotropic chiral or achiral anisotropic materials. These characteristics enable precise control of SPP propagation and polarization, allowing engineers and scientists to design tunable nanophotonic devices for advanced technological applications [17].

For tunable SPP propagation, the partnering material plays a vital role. Any chemical or physical change near the interface can alter the properties of SPPs, enabling optical and chemical sensing [18]. Similarly, if the material is temperature-sensitive, the interface can guide SPPs differently at different temperatures [19]. Recently, many researchers have reported tunable surface-wave propagation supported by InSb. For example, Mackay and Lakhtakia proposed a temperature-sensitive hyperbolic composite structure based on InSb and examined surface-wave propagation at the interface between a hyperbolic structure and an isotropic material. They reported that surface-wave properties can be tuned by varying temperature and the composition fill factor. In addition, InSb can be used to thermally tune metasurfaces and metamaterials in the THz frequency range [20]. InSb is widely used in infrared detectors for applications including forward-looking infrared (FLIR) and thermal imaging in civilian and military aircraft, infrared astronomy [21,22], and infrared homing missile guidance systems. 

InSb detectors are highly sensitive in the 1–5 µm wavelength range. Moreover, because InSb is a strong photo-Dember emitter, it can be employed as a source of THz radiation [23]. A layer of aluminum indium antimonide (AlInSb) can serve as a quantum well in high-speed electronics [24], ballistic-transport devices, spintronics, and other quantum applications [25]. In addition, AlInSb provides a practical strain-compensating barrier material for antimonide device structures and mid-infrared interband cascade lasers [24]. Although a broad propagation-frequency range is essential for many applications, temperature-dependent electromagnetic surface waves supported by InSb have so far been investigated only over a limited range of visible and infrared frequencies. To address this gap, this work proposes an InSb-UAC-InSb planar interface for temperature-dependent surface waves in the near-infrared frequency range. Indium antimonide (InSb), a narrow-bandgap semiconductor, has strong potential for FLIR systems, infrared homing missile-guidance systems, and infrared astronomy [23,26].

In this paper, we theoretically investigate surface plasmon (SP) modes in an InSb-UAC-InSb planar interface operating in the near-infrared spectral region. Unlike previously reported chiral waveguide structures based on metals or graphene, the proposed structure combines a temperature-sensitive InSb material with a uniaxial chiral medium, enabling simultaneous control of SPP characteristics through chirality and external temperature. This combination provides an additional degree of freedom for controlling dispersion and propagation behavior that is not available in conventional plasmonic waveguides.

## 2. Methodology

**Figure 1:**
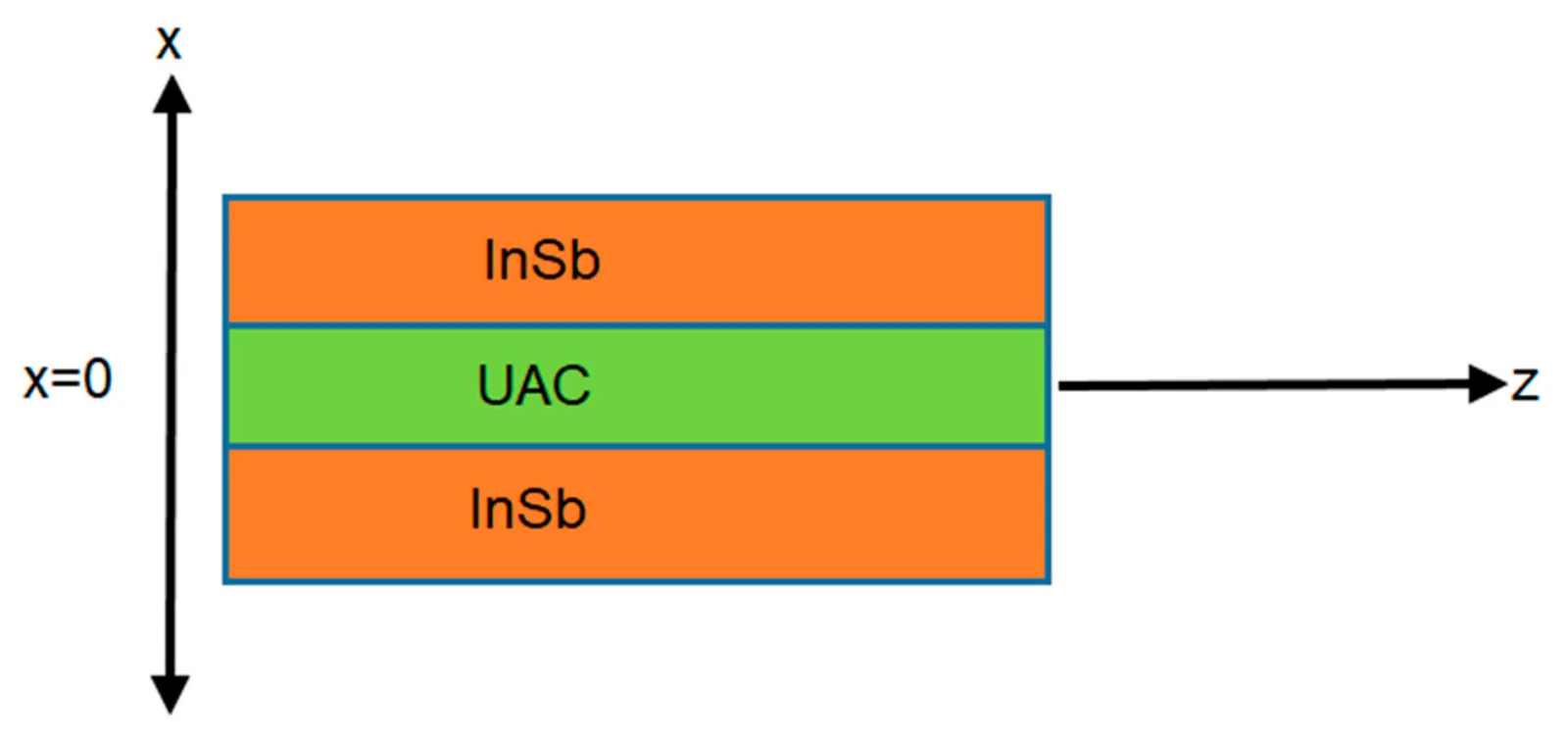
InSb-UAC-InSb geometry.

In this geometry, electromagnetic (EM) surface-wave propagation is investigated along the InSb-UAC-InSb planar interface. The EM surface wave is assumed to propagate along the *z*-axis and attenuate along the *x*-axis, as shown in Figure 1.

The EM field components for x > 0 are given by

(1) $$E_{z1}=A(e^{-\gamma_{1}x})$$

(2) $$H_{z1}=B(e^{-\gamma_{1}x})$$

(3) $$E_{y1}=\frac{i\omega \mu_{0}}{\gamma_{1}}B(e^{-\gamma_{1}x})$$

(4) $$H_{y1}=-\frac{i\omega \varepsilon_{0}}{\gamma_{1}}A(e^{-\gamma_{1}x})$$

Here, the symbols denote the decay constant, wave frequency, permittivity, and permeability of free space. The EM waves in the UAC medium are characterized by the following constitutive relations.

(5) D=εtI̿t+εze^ze^z.E−jξμ0ε0e^ze^z.HB=μtI̿t+μze^ze^z.H−jξμ0ε0e^ze^z.E

Here, ${\hat{e}}_{x}$, ${\hat{e}}_{y}$ and ${\hat{e}}_{z}$ are unit vectors that are mutually perpendicular to each other. The dyadic vector ${\overset{̿}{I}}_{t}={\hat{e}}_{x}{\hat{e}}_{x}$ + ${\hat{e}}_{y}{\hat{e}}_{y}$ describes the constitutive relation in the above equation. $\mu_{0}$ and $\varepsilon_{0}$ denote the permeability and permittivity of free space. $\mu_{t}{,\;\varepsilon }_{t}$ denote the transverse components and $\mu_{z},\varepsilon_{z}$ denote the longitudinal components of permeability and permittivity of the medium. ξ represents the chirality parameter which governs the coupling of electric and magnetic field components [27,28].

The field components in the UAC medium are given by

(6) $$E_{z2}=C(e^{{-q}_{1}x})+D(e^{q_{1}x})+E(e^{{-q}_{2}x})+F(e^{q_{2}x})$$

(7) $$H_{z2}=\frac{i\;\alpha_{1}}{\eta_{t}}[C(e^{{-q}_{1}x})+D(e^{q_{1}x})]+\frac{i\;\alpha_{2}}{\eta_{t}}[E(e^{{-q}_{2}x})+F(e^{q_{2}x})]$$

The remaining field components for the UAC medium can be obtained from [29].

(8) $${q_{1,2}}^{2}=\frac{\lambda^{2}}{2}\left[\frac{\mu_{z}}{\mu_{t}}+\frac{\varepsilon_{z}}{\varepsilon_{t}}\pm \sqrt{{\left(\frac{\mu_{z}}{\mu_{t}}-\frac{\varepsilon_{z}}{\varepsilon_{t}}\right)}^{2}+\frac{4{\xi^{2}\mu }_{z}\varepsilon_{z}}{\mu_{t}\varepsilon_{t}}}\right]$$

Here, $\lambda =\sqrt{\beta^{2}-\omega^{2}{\mu_{t}\varepsilon }_{t}}$ is the decay constant. β and ω represent the propagation constant and operating frequency, respectively, where $\alpha_{1}=\left(\frac{{k_{1}}^{2}}{\lambda^{2}}-\frac{\varepsilon_{z}}{\varepsilon_{t}}\right)\;\frac{\sqrt{{\mu_{t}\varepsilon }_{t}}}{\xi \;\sqrt{{\mu_{z}\varepsilon }_{z}}}$, $\alpha_{2}=\left(\frac{{k_{2}}^{2}}{\lambda^{2}}-\frac{\varepsilon_{z}}{\varepsilon_{t}}\right)\frac{\sqrt{{\mu_{t}\varepsilon }_{t}}}{\xi \;\sqrt{{\mu_{z}\varepsilon }_{z}}}$, and $\eta_{t}=\sqrt{\varepsilon_{t}/\mu_{t}}$.

The field components for x < 0 are given by

(9) $$E_{z3}=G(e^{\gamma_{1}x})$$

(10) $$H_{z3}=H(e^{\gamma_{1}x})$$

(11) $$E_{y3}=\frac{i\omega \mu_{0}}{\gamma_{1}}H(e^{\gamma_{1}x})$$

(12) $$H_{y3}=-\frac{i\omega \varepsilon_{0}}{\gamma_{1}}G\left(e^{\gamma_{1}x}\right)$$

Here, A, B, C, D, E, F, G, and H are unknown coefficients corresponding to the EM-field amplitudes. These coefficients are obtained by applying the appropriate boundary conditions. $\gamma_{1}=\sqrt{\beta^{2}-\omega^{2}\varepsilon_{InSb}\mu_{0}}$ is the decay constant and $k_{0}=\omega \sqrt{\mu_{0}\varepsilon_{0}}$. In the near-infrared regime, InSb is assumed to be a nonmagnetic material; therefore, its permeability is obtained from [30]. InSb is selected as the thermally tunable medium because its electromagnetic properties are highly temperature-sensitive. In the frequency range considered in this work, the complex permittivity of InSb is obtained using the Drude model [31,32].

(13) $$\varepsilon_{InSb}=\varepsilon_{\infty }-\frac{\omega_{p}^{2}}{\omega^{2}+\mathrm{i}\gamma \omega }\;$$

where $\omega_{p}=\sqrt{Nq_{e}^{2}/0.015\varepsilon_{0}m}$ is the plasma frequency,$\;q_{e}=-1.60\times 10^{-19}C$ is the electron charge, and $m=9.11\times 10^{-31}\;kg\;is\;the\;mass\;ofelectron.$ The Drude permittivity incorporates InSb material loss through the damping constant, which is given by $\gamma =\pi \times 10^{11}\;rad\;s^{-1},$ and high-frequency relative permittivity is $\varepsilon_{\infty }=15.68$. The temperature dependence of $\varepsilon_{InSb}$ is incorporated through the intrinsic carrier density relation, i.e., $N=5.76\times 10^{20}{T\;}^{\frac{3}{2}}exp(-\frac{E_{g}}{2K_{B}T})$, where $E_{g}$ represents the band gap, whose value is $E_{g}=0.26\;eV$, and $K_{B}$ denotes the Boltzmann constant $K_{B}=8.62\times 10^{-5}\;eV\;K^{-1}$ [33,34].

(14) $$\hat{x}\times \left[H_{1}-H_{2}\right]=0$$

(15) $$\hat{x}\times \left[E_{1}-E_{2}\right]=0$$

These boundary conditions are applied to obtain the following characteristic equation:

(16) $$\left[\begin{array}{cccccccc} a_{11} & a_{12} & a_{13} & a_{14} & a_{15} & a_{16} & a_{17} & a_{18}\\ a_{21} & a_{22} & a_{23} & a_{24} & a_{25} & a_{26} & a_{27} & a_{28}\\ a_{31} & a_{32} & a_{33} & a_{34} & a_{35} & a_{36} & a_{37} & a_{38}\\ a_{41} & a_{42} & a_{43} & a_{44} & a_{45} & a_{46} & a_{47} & a_{48}\\ a_{51} & a_{52} & a_{53} & a_{54} & a_{55} & a_{56} & a_{57} & a_{58}\\ a_{61} & a_{62} & a_{63} & a_{64} & a_{65} & a_{66} & a_{67} & a_{68}\\ a_{71} & a_{72} & a_{73} & a_{74} & a_{75} & a_{76} & a_{77} & a_{78}\\ a_{81} & a_{82} & a_{83} & a_{84} & a_{85} & a_{86} & a_{87} & a_{88} \end{array}=0\right]$$

$$\begin{array}{c} a_{11}=0,\;a_{12}=\eta_{t},\;a_{13}=-\alpha_{1},\;a_{14}=-e^{2dq_{1}}\alpha_{1},\;a_{15}=-\alpha_{2},\;a_{16}=-e^{2dq_{2}}\alpha_{2},\;a_{17}=0,\;a_{18}=0,\\ a_{21}=-\frac{i\varepsilon \eta_{t}\omega }{\gamma_{1}},\;a_{22}=0,\;a_{23}=-\frac{k_{t}{\alpha_{1}}^{2}}{q_{1}},\;a_{24}=\frac{e^{2dq_{1}}k_{t}{\alpha_{1}}^{2}}{q_{1}},\;a_{25}=-\frac{k_{t}{\alpha_{2}}^{2}}{q_{2}},\;a_{26}=\frac{e^{2dq_{2}}k_{t}{\alpha_{2}}^{2}}{q_{2}},\;a_{27}=0,\\ a_{28}=0,\;a_{31}=0,\;a_{32}=-\frac{\mu_{0}\omega }{k_{t}\gamma_{1}},\;a_{33}=-\frac{\alpha_{1}}{q_{1}},\;a_{34}=\frac{e^{2dq_{1}}\alpha_{1}}{q_{1}},\;a_{35}=-\frac{\alpha_{2}}{q_{2}},\;a_{36}=\frac{e^{2dq_{2}}\alpha_{2}}{q_{2}},\;a_{37}=0,\\ a_{38}=0,\;a_{41}=i,\;a_{42}=0,\;a_{43}=-1,\;a_{44}=-e^{2dq_{1}},\;a_{45}=-1,\;a_{46}=-e^{2dq_{2}},\;a_{47}=0,\;a_{48}=0,\\ a_{51}=0,\;a_{52}=0,\;a_{53}=e^{2dq_{1}}\alpha_{1},\;a_{54}=\alpha_{1},\;a_{55}=e^{2dq_{2}}\alpha_{2},\;a_{56}=\alpha_{2},\;a_{57}=0,\;a_{58}=-\eta_{t},\\ a_{61}=0,\;a_{62}=0,\;a_{63}=\frac{e^{2dq_{1}}{\alpha_{1}}^{2}}{q_{1}},\;a_{64}=-\frac{{\alpha_{1}}^{2}}{q_{1}},\;a_{65}=\frac{e^{2dq_{2}}{\alpha_{2}}^{2}}{q_{2}},\;a_{66}=-\frac{{\alpha_{2}}^{2}}{q_{2}},\;a_{67}=-\frac{i\varepsilon \eta_{t}\omega }{k_{t}\gamma_{1}},\;a_{68}=0,\\ a_{71}=0,\;a_{72}=0,\;a_{73}=\frac{e^{2dq_{1}}k_{t}\alpha_{1}}{q_{1}},\;a_{74}=-\frac{k_{t}\alpha_{1}}{q_{1}},\;a_{75}=\frac{e^{2dq_{2}}k_{t}\alpha_{2}}{q_{2}},\;a_{76}=-\frac{k_{t}\alpha_{2}}{q_{2}},\;a_{77}=0,\;a_{78}=-\frac{\mu_{0}\omega }{\gamma_{1}},\\ a_{81}=0,\;a_{82}=0,\;a_{83}=e^{2dq_{1}},\;a_{84}=1,\;a_{85}=e^{2dq_{2}},\;a_{86}=1,\;a_{87}=-i,\;a_{88}=0 \end{array}$$

The analytical and numerical results are analyzed using the characteristic equation in Equation (16).

## 3. Results and Discussion

In this section, numerical analysis is conducted to evaluate the impact of material parameters on the properties of electromagnetic (EM) surface waves along the InSb-UAC-InSb planar interface using the characteristic equation in Equation (16). For the EM surface-wave analysis, dispersion curves are plotted for different values of chirality, core-width, InSb temperature, and operating frequency for two cases of the uniaxial chiral medium. These two cases provide an additional degree of freedom for tunable SPPs. In UAC media, the transverse and longitudinal permittivities may be positive or negative depending on the structural configuration and operating frequency [28,35,36]. To simplify the realization of the UAC medium, a consistent assumption is adopted throughout the analysis. To ensure stable solutions of the characteristic equation, numerical calculations were performed using Wolfram Mathematica with controlled numerical precision and convergence criteria. The numerical stability of the calculated dispersion results was verified by checking that further changes to the computational settings did not result in appreciable variations in the reported results.

**Case I:** $\varepsilon_{t}>0,\varepsilon_{z}<0$

**Figure 2:**
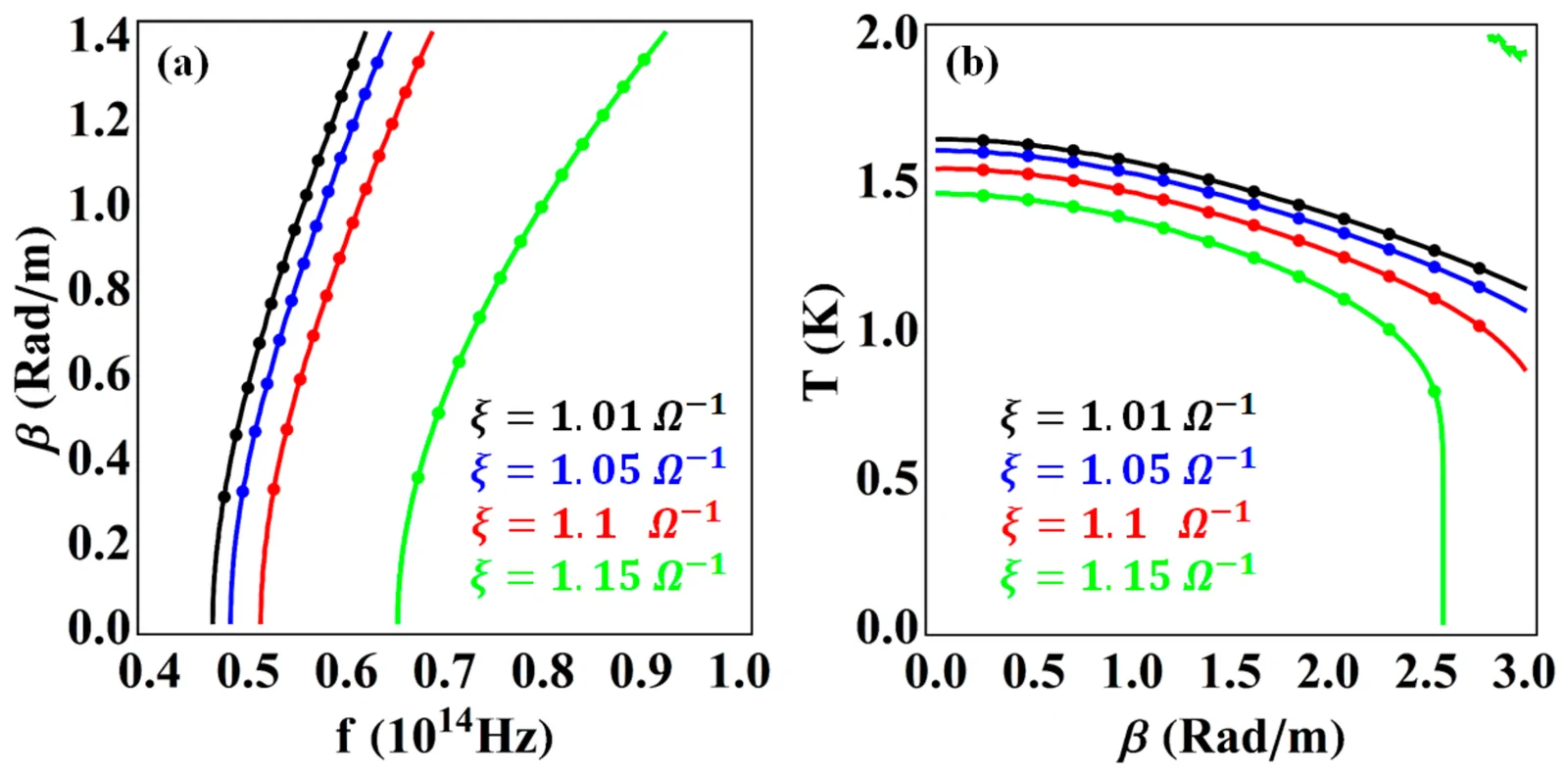
(**a**) Variation in the propagation constant as a function of operating frequency for different chirality values and (**b**) variation in the propagation constant as a function of InSb temperature for different chirality values.

**Figure 3:**
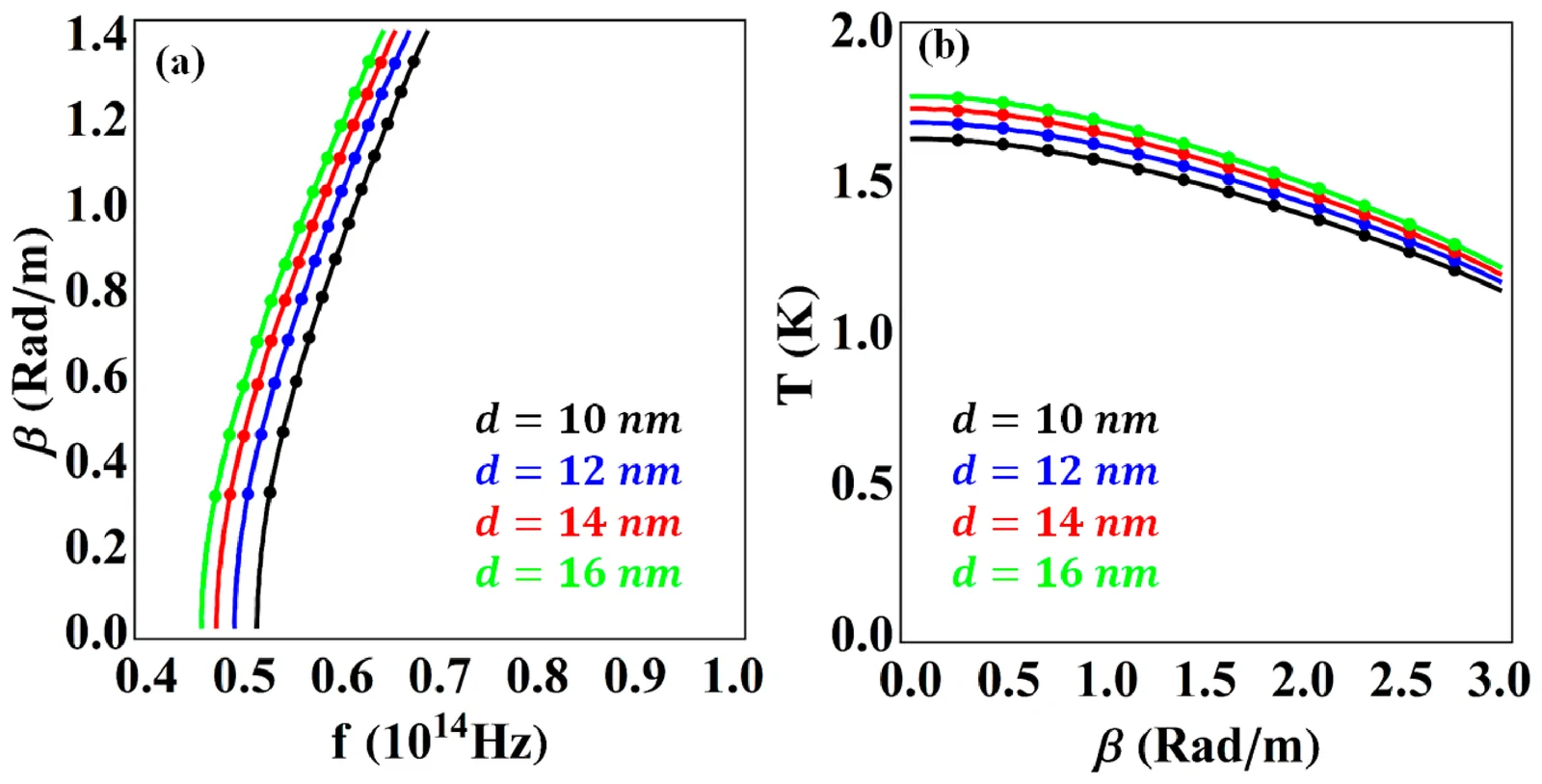
(**a**) Variation in the propagation constant as a function of operating frequency for different core-width values and (**b**) variation in the propagation constant as a function of InSb temperature for different core-width values.

In this case, the transverse and longitudinal permittivities are selected to analyze the propagation constant, consistent with the literature showing that these parameters may be either positive or negative [35]. Figure 2a shows the effect of chirality on the propagation constant as a function of incident-wave frequency. As the chirality parameter increases, the propagation constant increases with frequency, indicating that optical activity can be used to tune the plasmon mode [15,37,38]. The propagation constant $\left(\beta \right)$ increases with chirality due to the stronger magnetoelectric coupling introduced by the chiral constitutive terms. This coupling modifies the hybridization of the transverse electric (TE) and transverse magnetic (TM) components and alters the allowed propagation constant obtained from the characteristic equation [39]. A sharp increase in the propagation constant indicates strong field confinement and reduced phase velocity. The influence of chirality on the propagation constant as a function of InSb temperature is shown in Figure 2b. The propagation constant decreases with temperature as the chirality value increases. The temperature-dependent variation in the propagation constant results from the modification of the InSb dielectric response through the temperature-dependent carrier concentration, resulting in different dispersion responses for different chirality values. Lower chirality values exhibit higher propagation constants at higher temperatures. A key advantage of using a UAC medium is that the degree of chirality can be significantly controlled, which is difficult in the resulting change in permittivity modifies. To further study surface-wave confinement in the InSb-UAC-InSb structure, the effect of core-width on the propagation constant as a function of incident-wave frequency is shown in Figure 3a. Increasing the core-width increases the propagation constant at a given operating frequency. Figure 3a shows that the core-width plays an important role in tuning SPP propagation. As the core-width increases, the propagation constant also increases as the frequency decreases; consequently, the confinement of these waves decreases at higher frequencies. The effect of core-width on the propagation constant versus InSb temperature is shown in Figure 3b. Changing the core-width alters the spatial overlap of the modal fields with the interfaces, thereby altering the coupling between interface-supported modes. This modifies the propagation constant and produces the observed shift in temperature-dependent dispersion curves. These trends are consistent with the findings reported in [14], which confirmed that the propagation constant is highly sensitive to core-width. Thus, the present results extend the framework to the InSb-based near-infrared regime and confirm the tunability of the dispersion characteristics. The proposed structure provides additional tunability because of the temperature sensitivity of InSb.

**Figure 4:**
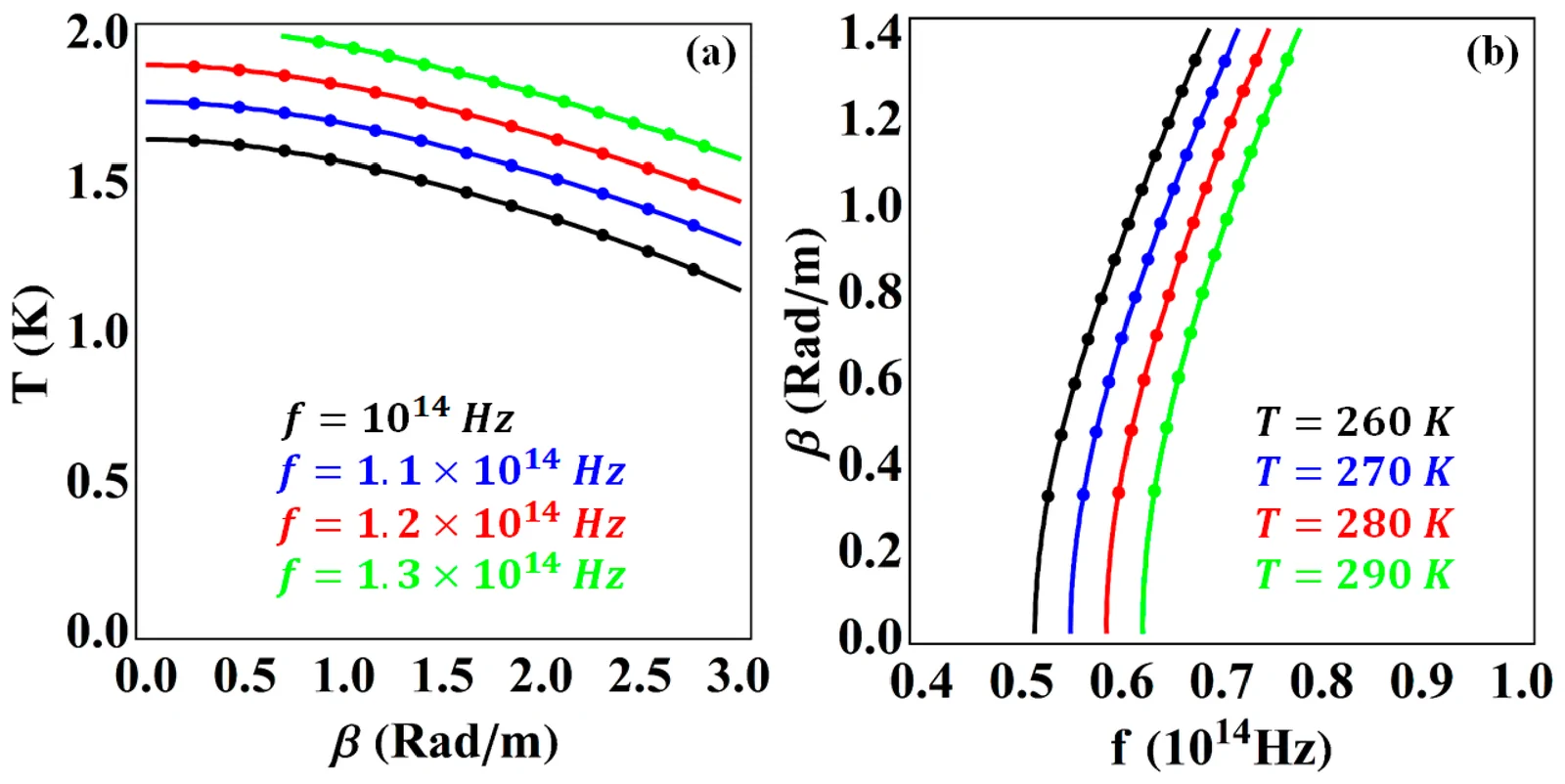
(**a**) Variation in the propagation constant as a function of InSb temperature for different frequency values and (**b**) variation in the propagation constant as a function of operating frequency for different InSb temperatures.

The influence of different operating frequencies in the near-infrared region on the propagation constant as a function of InSb temperature is shown in Figure 4a. Higher operating frequencies shift the curves upward, indicating strong field confinement. Furthermore, the temperature is higher at lower propagation-constant values but decreases significantly as the propagation constant increases. Figure 4b shows the temperature dependence of the dispersion relation. The effect of InSb temperature is analyzed by plotting the propagation constant against incident-wave frequency. As the temperature increases from 260 K to 290 K, the propagation-constant curves shift toward higher frequencies, which can be attributed to the strongly temperature-dependent dielectric function of InSb. The temperature tunability of the proposed structure originates from the temperature dependence of the intrinsic carrier concentration of InSb [40]. This behavior is consistent with earlier studies on thermally tunable surface waves in InSb-based systems [20,41], where the carrier concentration changes with temperature, modifying the plasma frequency according to $\omega_{p}=\sqrt{Nq_{e}^{2}/0.015\varepsilon_{0}m}$. The plasma frequency enters directly into the Drude permittivity; the resulting change in permittivity modifies the transverse decay constant and the characteristic equation governing the SPP mode. These findings confirm that both frequency and temperature provide effective control for tailoring the plasmon mode at the InSb-UAC-InSb interface. This temperature sensitivity of InSb can be utilized in thermally tunable metasurfaces, metamaterials, thermo-optical sensing devices, and thermally reconfigurable plasmonic components [20].

**Figure 5:**
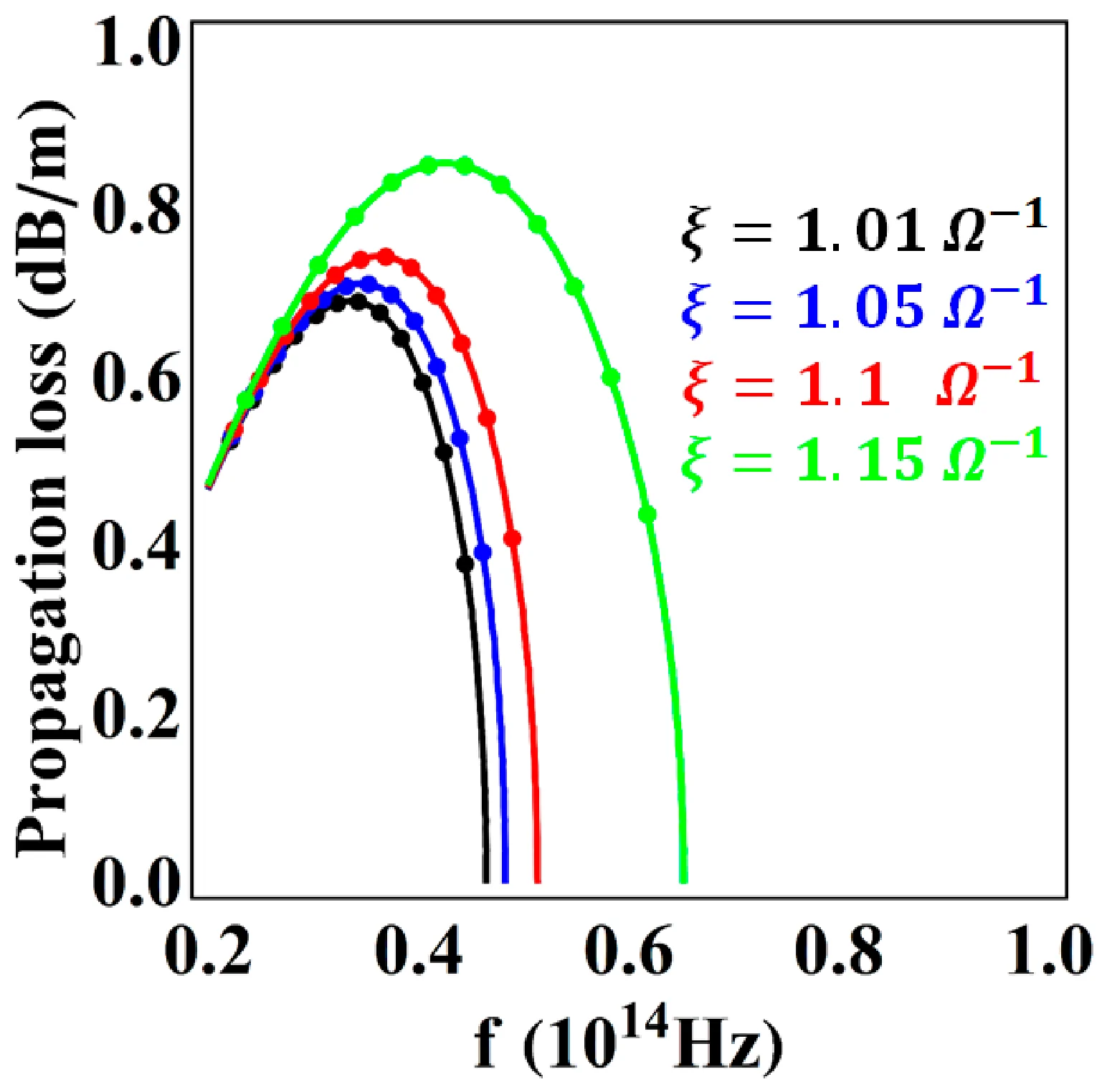
Variation in the propagation loss as a function of operating frequency under different chirality values.

The effect of chirality on propagation loss as a function of operating frequency is shown in Figure 5. Initially, the propagation loss increases with frequency and reaches a maximum value; after a certain frequency, the propagation loss decreases for all considered chirality values. Higher chirality values lead to higher propagation loss and shift the curves toward higher-frequency regime. The chirality parameter enters through magnetoelectric coupling in the constitutive relation and directly influences the electromagnetic dispersion. This changes the modal field distribution and its interaction with the lossy InSb regions. Consequently, the increase in propagation loss with chirality indicates stronger modal dissipation within the investigated frequency range. The result demonstrates that chirality provides additional degrees of freedom for controlling the propagation loss of the guided SPP mode [37].

**Case II:** $\varepsilon_{t}<0,\varepsilon_{z}>0$

**Figure 6:**
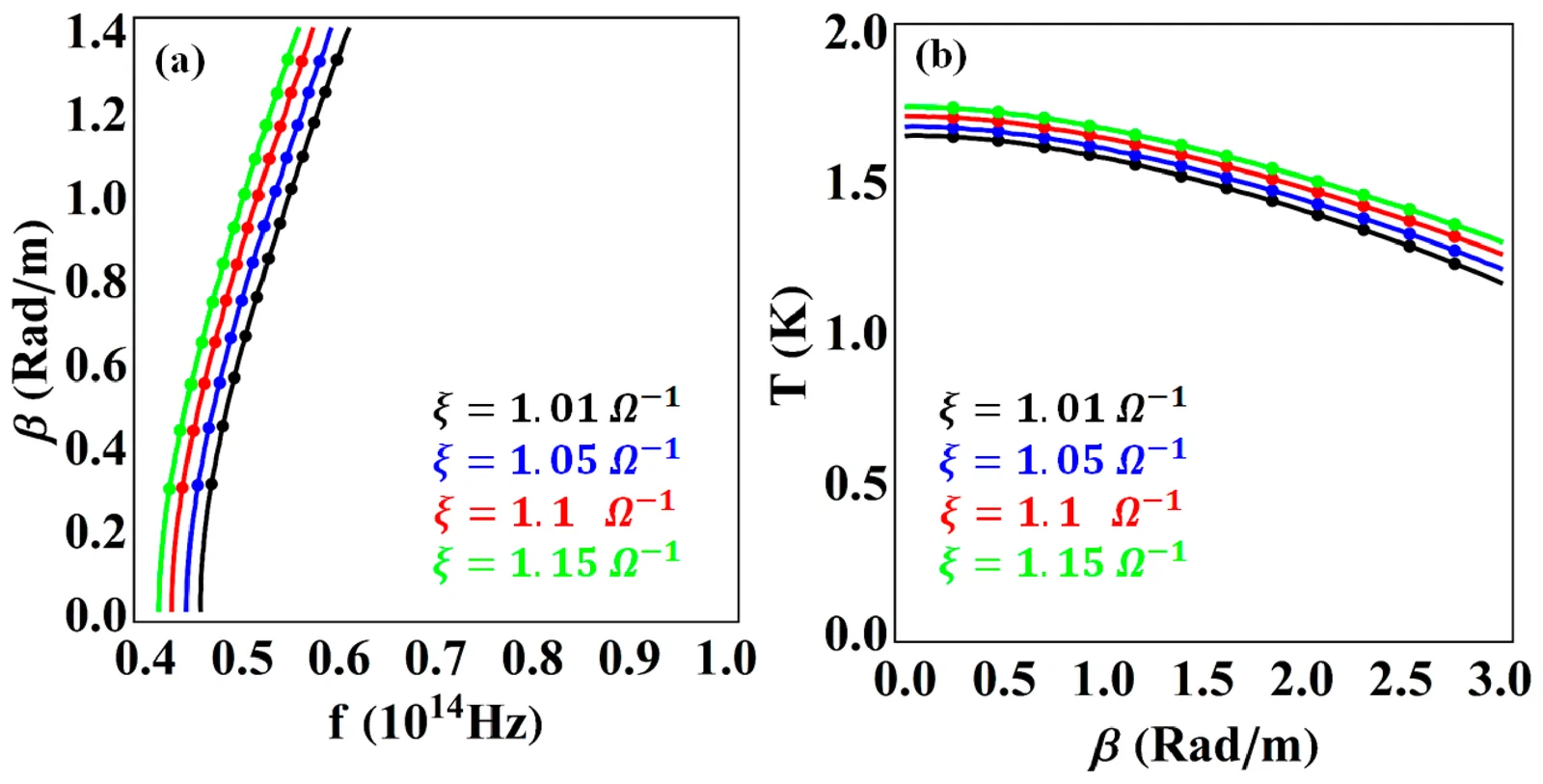
(**a**) Variation in the propagation constant as a function of operating frequency for different chirality values and (**b**) variation in propagation constant as a function of InSb temperature for different chirality values.

**Figure 7:**
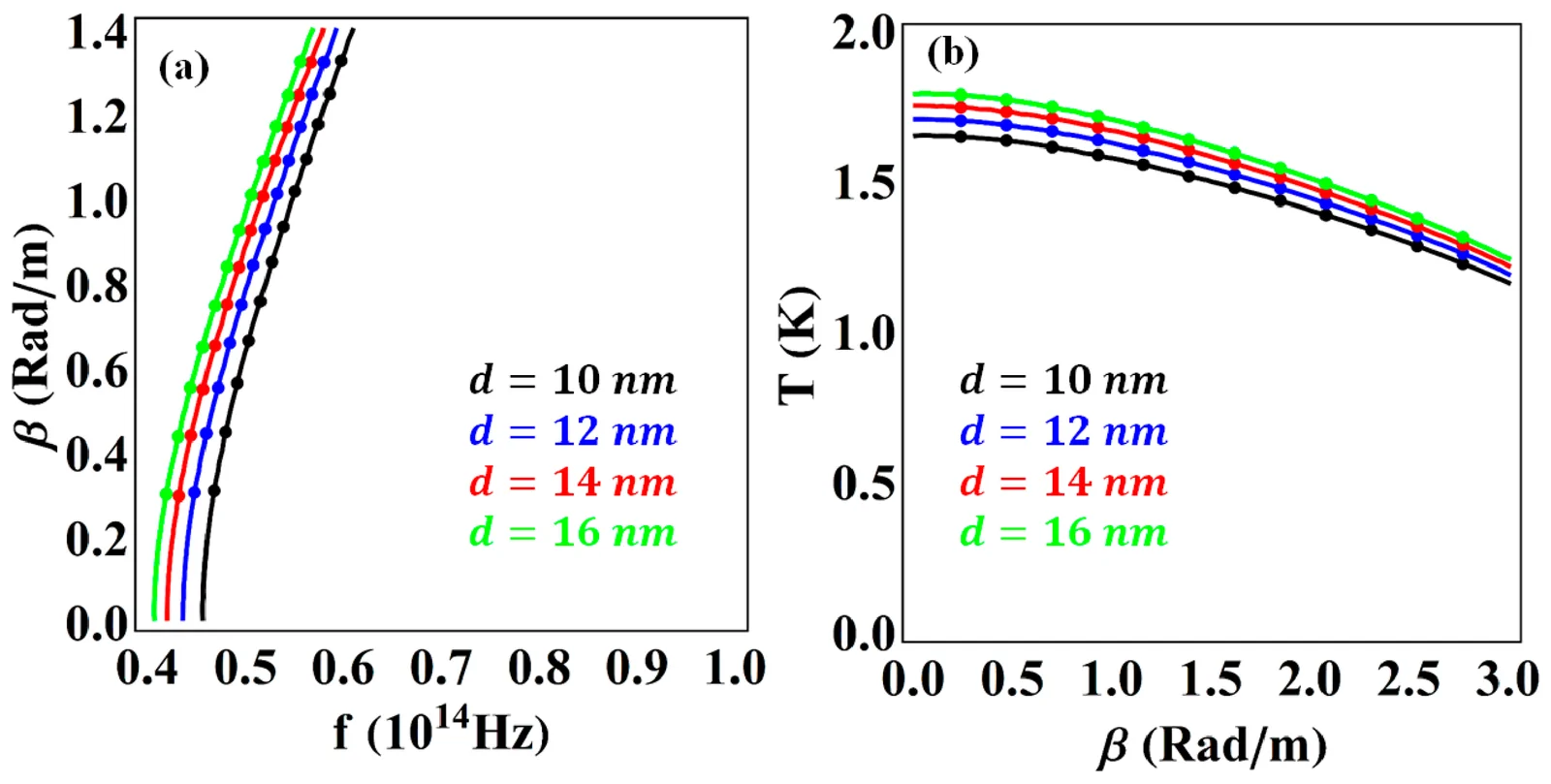
(**a**) Variation in the propagation constant as a function of operating frequency for different core-width values and (**b**) variation in the propagation constant as a function of InSb temperature for different core-width values.

In this case, the transverse and longitudinal permittivities are selected for the analysis of the propagation constant. The influence of the chirality parameter on the propagation constant versus incident-wave frequency in the near-infrared region is shown in Figure 6a. The propagation constant is plotted against operating frequency for four different chirality values. Increasing the chirality value results in a consistent upward shift in the dispersion curves, indicating enhanced SPP propagation. As the chirality value increases, the propagation constant increases at lower operating frequencies. The propagation constant is plotted as a function of InSb temperature for the same chirality values in Figure 6b. Although temperature decreases monotonically with increasing propagation constant, higher chirality values lead to higher propagation constants. These results confirm that chirality is an effective parameter for tuning plasmon modes, which may be useful for tunable photonic components in the near-infrared region [42]. Figure 7a shows the effect of core-width on the propagation constant versus incident-wave frequency. The propagation constant increases with decreasing operating frequency as the core-width increases. The curves remain close to each other, showing that the effect of core-width is moderate but not negligible in this frequency regime. In Figure 7b, the effect of core-width on the propagation constant versus InSb temperature is analyzed. The propagation constant increases with increasing core-width. Larger core-widths exhibit higher temperature values; however, at higher propagation constants, the temperature curves shift toward lower values. 

**Figure 8:**
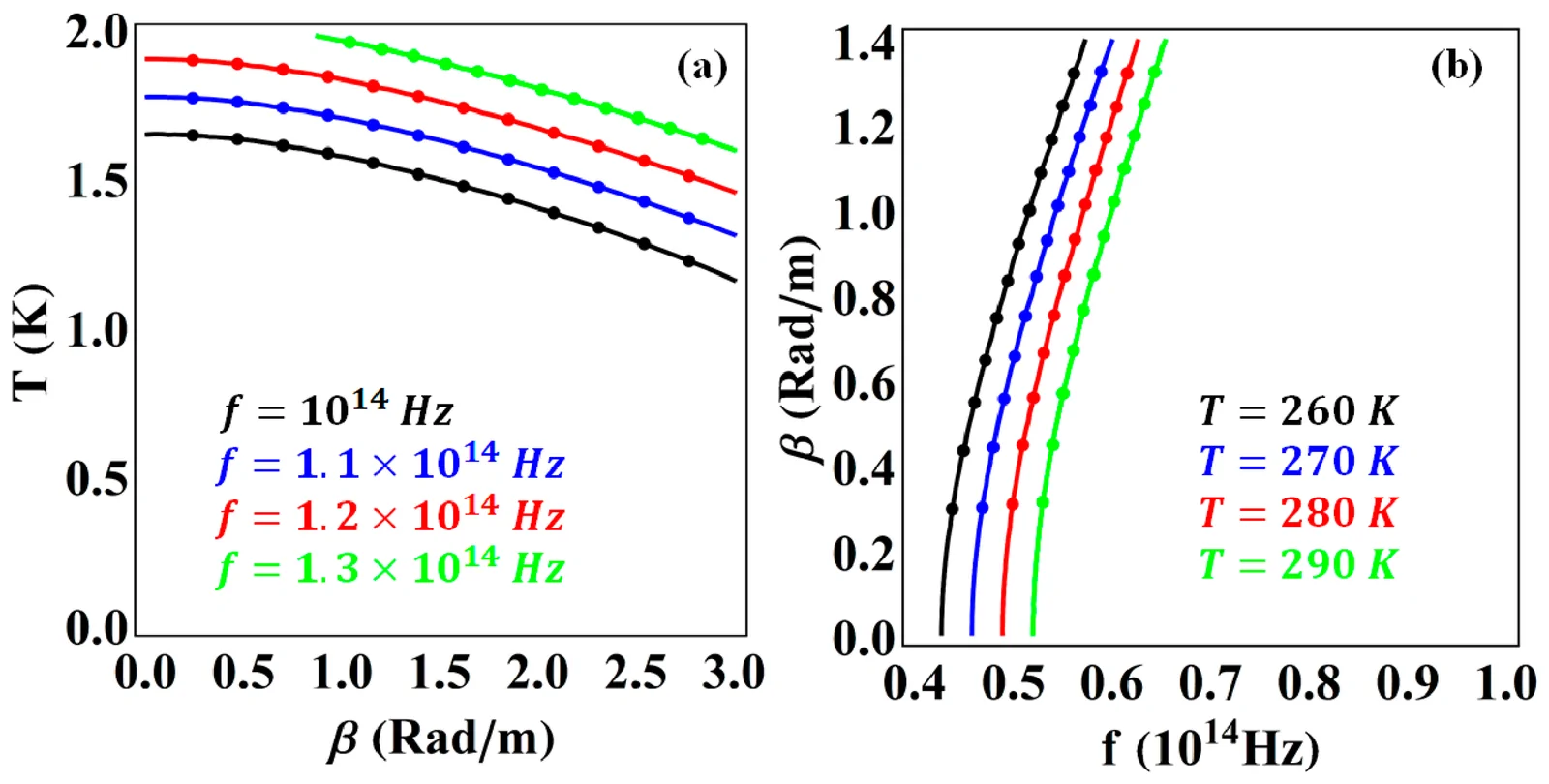
(**a**) Variation in the propagation constant as a function of InSb temperature for different frequency values and (**b**) variation in propagation constant as a function of operating frequency for different InSb temperatures.

The impact of operating frequency on the propagation constant versus InSb temperature is analyzed in Figure 8a. InSb temperature decreases monotonically with increasing propagation constant as the operating frequency varies. Higher frequencies correspond to larger temperature values, indicating enhanced coupling at high frequencies. The curve shift demonstrates the sensitivity of the proposed structure to frequency tuning in the near-infrared region. The influence of InSb temperature on the propagation constant versus incident-wave frequency is shown in Figure 8b. As the InSb temperature varies from 260 K to 290 K, the propagation constant increases with increasing incident-wave frequency. Even small variations in temperature can significantly affect the permittivity [40]. At higher temperatures, the carrier concentration in InSb is increases, and by varying the carrier concentration, the plasma frequency of InSb can be tuned [43,44]. The tunability of SPPs through temperature and frequency may support the design of surface waveguides and innovative thermo-optical sensors in the near-infrared spectral region [33].

**Figure 9:**
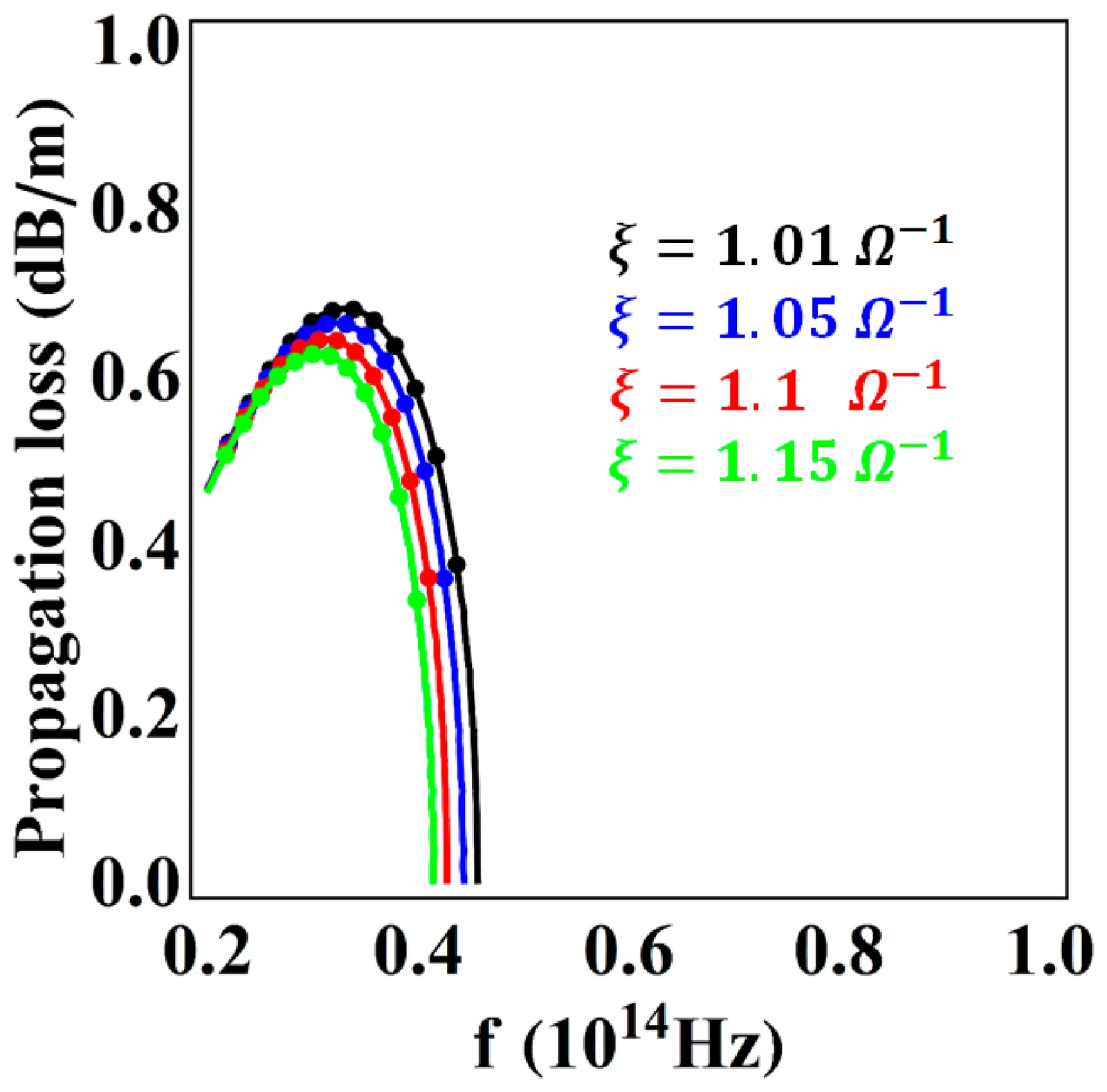
Variation in the propagation loss as a function of operating frequency under different chirality values.

The influence of chirality on propagation loss as a function of operating frequency is shown in Figure 9. Initially, the propagation loss increases with frequency and reaches a maximum value; it then begins to decrease, indicating the cutoff frequency. Unlike conventional chiral plasmonic behavior, the results show that increasing chirality decreases propagation loss. As the chirality value increases, the propagation loss decreases, and the curves shift toward the lower-frequency region. This result indicates that higher chirality reduces energy dissipation in the system, resulting in more efficient SPP propagation. At higher chirality values, the propagation loss remains relatively low, confirming the potential of the proposed geometry for low-loss plasmonic applications. The results demonstrate that chirality can effectively tune the propagation constant and reduce propagation loss compared with previously reported nonchiral–metal interfaces and graphene-based plasmonic devices, where losses are typically dominated by intrinsic material absorption [6,45]. Thus, the proposed geometry allows for tunable plasmonic characteristics and provides an additional degree of freedom because of the temperature sensitivity of InSb and the chirality of the UAC medium.

**Figure 10:**
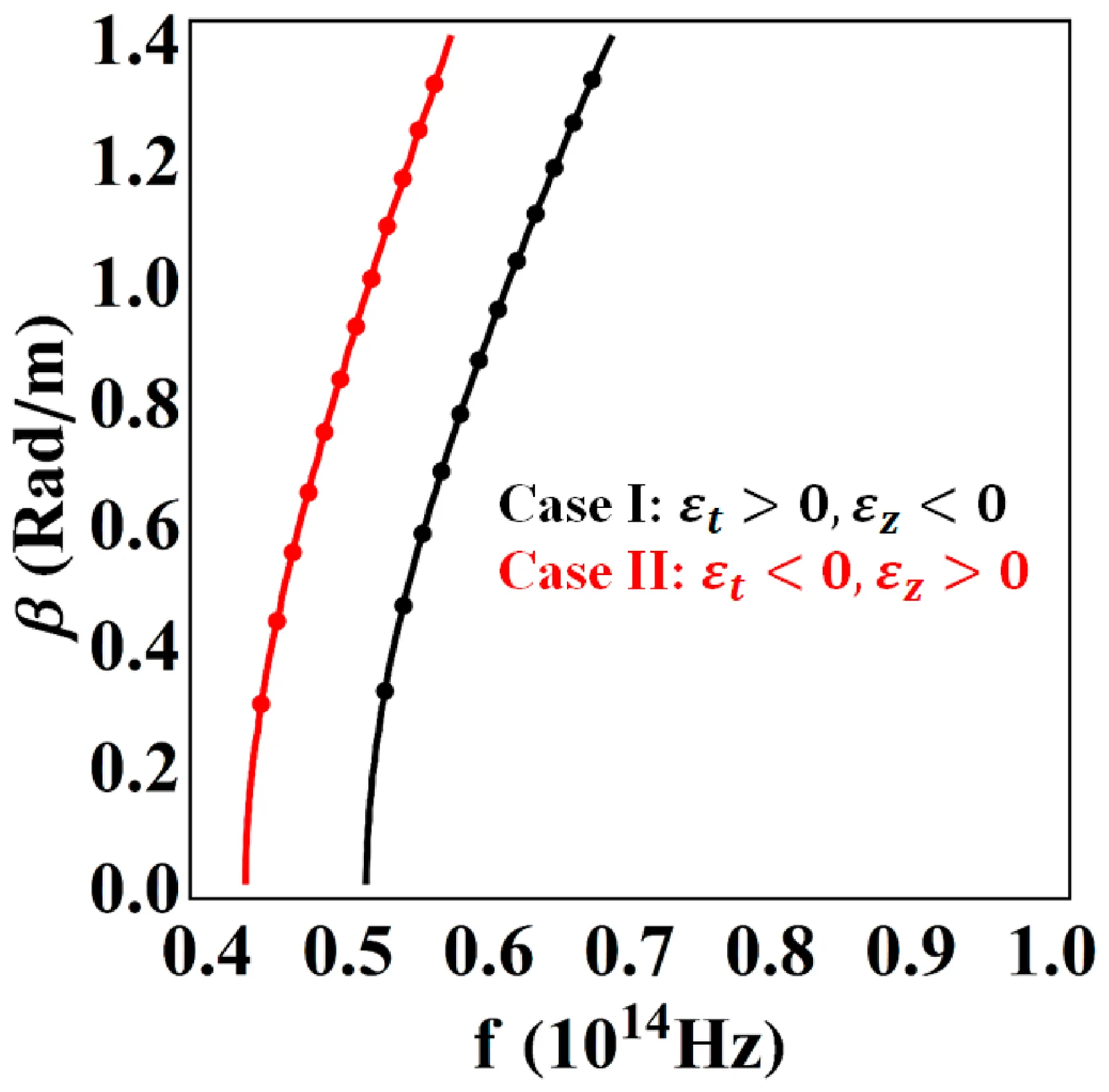
Comparison of the propagation constant as a function of operating frequency for Case I ${(\varepsilon }_{t}>0,\varepsilon_{z}<0)$ and Case II ${(\varepsilon }_{t}<0,\varepsilon_{z}>0)$ of the InSb-UAC-InSb waveguide.

**Figure 11:**
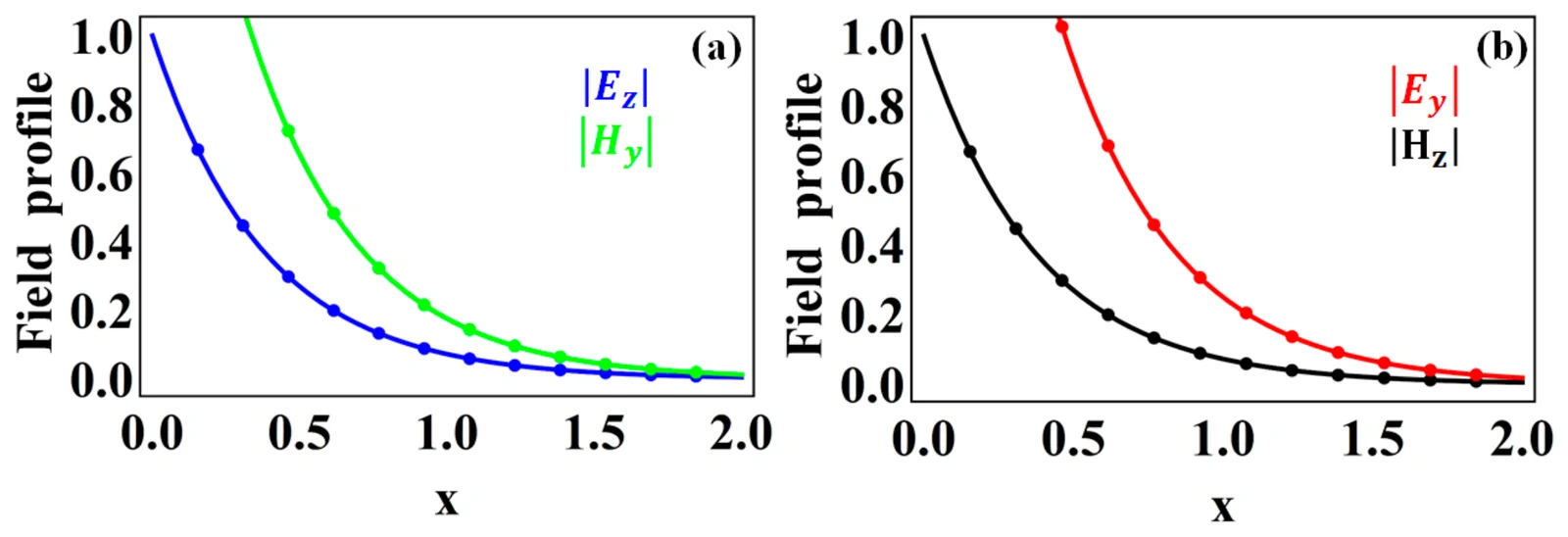
Normalized electromagnetic field profiles of the InSb-UAC-InSb structure (**a**) $\left|E_{z}\right|$ and $\left|H_{y}\right|$ and (**b**) $\left|E_{y}\right|$ and $\left|H_{z}\right|$.

**Figure 12:**
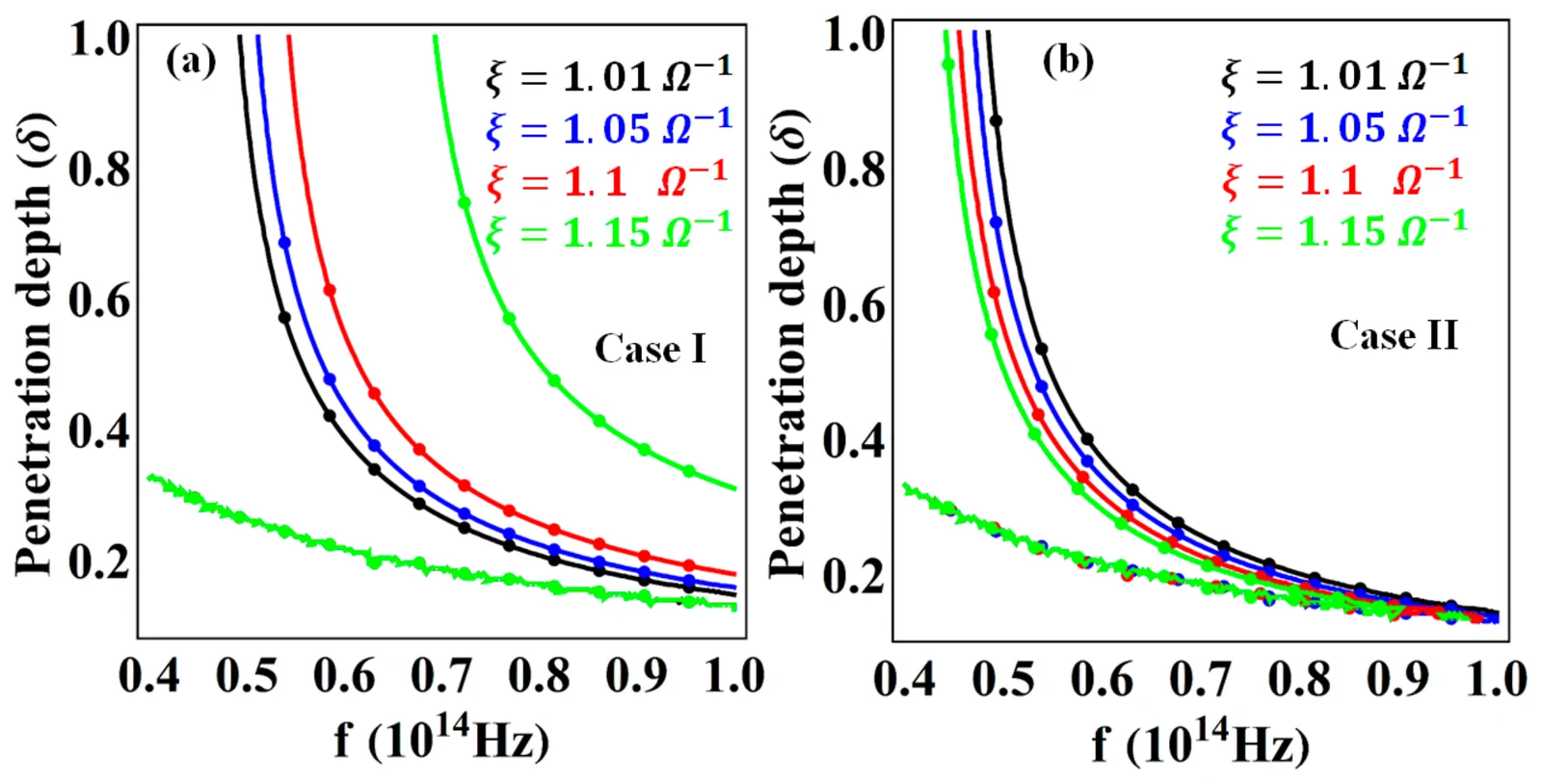
Penetration depth of the SPP mode as a function of operating frequency for different chirality values (**a**) Case I ${(\varepsilon }_{t}>0,\varepsilon_{z}<0)$ and (**b**) Case II ${(\varepsilon }_{t}<0,\varepsilon_{z}>0)$.

**Figure 13:**
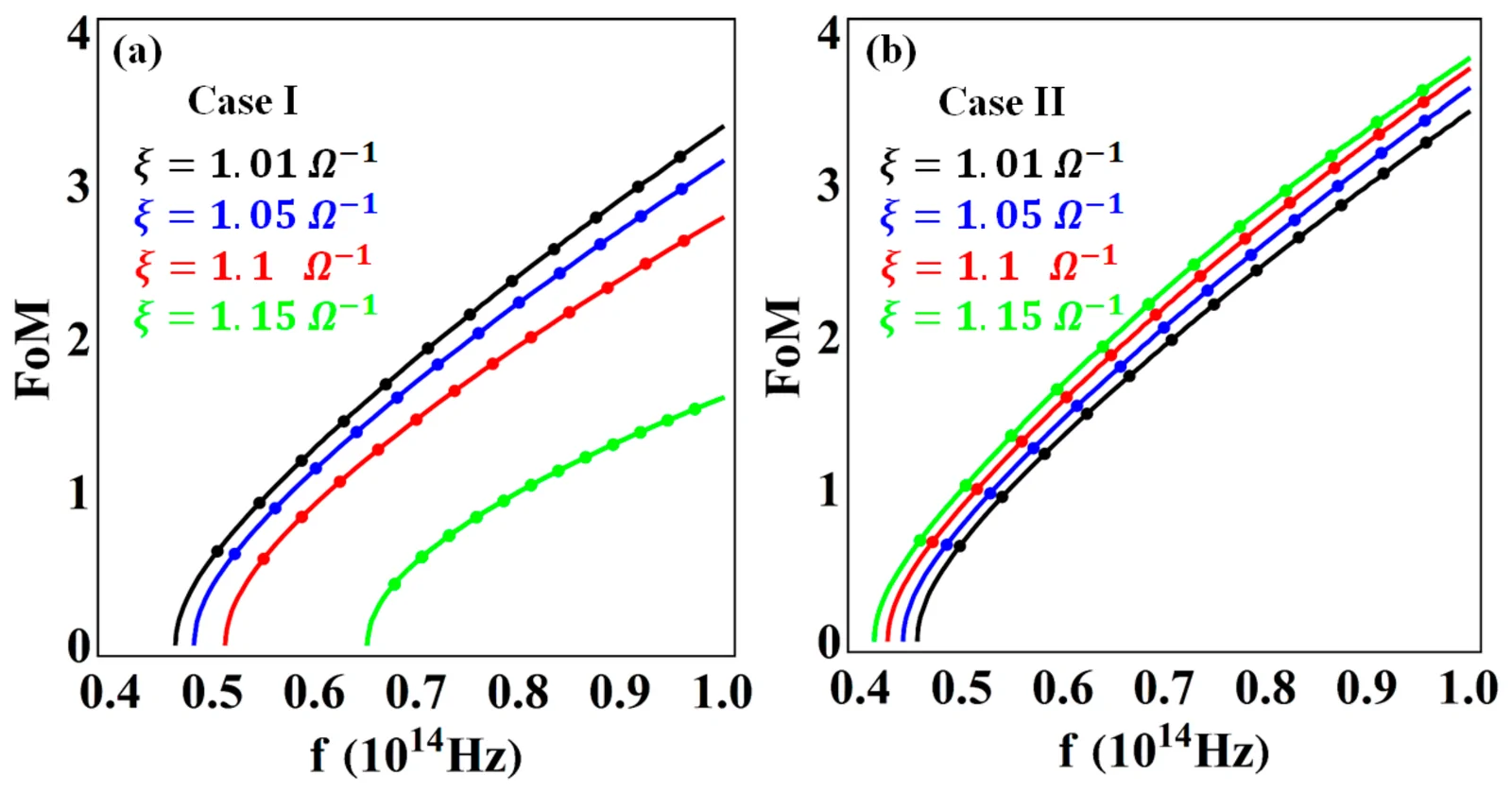
Figure of merit (FoM) of the SPP mode as a function of operating frequency for different chirality values (**a**) Case I ${(\varepsilon }_{t}>0,\varepsilon_{z}<0)$ and (**b**) Case II ${(\varepsilon }_{t}<0,\varepsilon_{z}>0).$

Figure 10 demonstrates the behavior of the propagation mode for the two cases of the UAC medium. In Case I (black curve), the propagation constant increases gradually with frequency, indicating a relatively broad frequency range for stable guided modes. In Case II (red curve), the propagation constant increases more sharply at low frequencies, showing that the structure is highly sensitive even at low frequencies. This sensitivity implies stronger confinement and higher field localization, making Case II more suitable for applications that require strong light-matter interaction. However, Case I offers smoother dispersion and may be more suitable for broadband waveguiding applications. The overall comparison shows that anisotropy plays a crucial role in tuning propagation characteristics, while Case II provides stronger confinement and higher sensitivity to frequency variation. Figure 11 presents the normalized field profiles to further verify the surface-wave nature and evaluate the spatial confinement of the supported electromagnetic mode. Figure 11a shows the normalized $E_{z}$ and $H_{y}$ components, whereas Figure 11b shows the normalized $E_{y}$ and $H_{z}$ components. The field components exhibit an exponential decay with increasing x, which indicates that electromagnetic energy is localized near the interface and decreases rapidly away from the interface. This spatial decay is a characteristic feature of a bound electromagnetic surface mode and confirms the confined nature of the supported SPP mode [46]. Moreover, the different decay rates in the electric and magnetic field components indicate that the mode has a hybrid character as a result of coupling between TE- and TM-like field components in the uniaxial chiral medium. To further evaluate the spatial confinement of the supported surface modes, the penetration depth is analyzed as a function of frequency for different chirality values in Figure 12. The penetration depth has an inverse relationship with the real part of the transverse decay constant and represents the characteristic distance over which the electromagnetic field decays away from the interface. Consequently, a smaller penetration depth corresponds to a stronger spatial confinement of the electromagnetic mode. Figure 12a shows that, for Case I, the penetration depth decreases with increasing frequency, indicating that the supported mode becomes increasingly localized near the interface at higher frequencies. Furthermore, the penetration depth is influenced by the chirality parameter, suggesting that chirality plays a significant role in controlling the spatial confinement of surface modes. As the chirality parameter increases, the penetration depth increases at a given operating frequency, indicating a reduction in spatial confinement for higher chirality values. Additionally, as the chirality parameter increases, the lower-frequency edge of the supported propagation region shifts to higher frequencies. As a result of this behavior, it is clear that chirality affects not only the propagation constant but also the spatial decay characteristics and the frequency range in which surface modes can propagate. Figure 12b illustrates a similar frequency-dependent reduction in penetration depth for Case II. As the penetration depths over a substantial portion of the investigated frequency range are relatively small, there is strong spatial localization of the electromagnetic field. Furthermore, the curves shift toward the lower-frequency region as the chirality value increases. Cases I and II exhibit different penetration-depth responses because their anisotropic permittivity configurations affect the modal decay constants, thereby altering the spatial distribution of electromagnetic fields. Combined with the normalized field profiles in Figure 11, these results provide quantitative evidence for the support of spatially confined electromagnetic surface modes in the proposed InSb-UAC-InSb structure. Further quantitative evaluation of the trade-off between modal confinement and propagation loss is performed using the figure of merit (FoM) of the supported SPP modes for both UAC configurations, as shown in Figure 13. FoM is defined as the ratio of the propagation length to the penetration depth. A higher FoM therefore indicates that the mode is capable of propagating over a greater range of penetration depths, corresponding to a more favorable combination of propagation and transverse localization. According to Figure 13a, the FoM for Case I increases with increasing operating frequency, indicating an improved propagation-to-confinement ratio in the high-frequency region of the investigated band. In contrast, increasing the chirality parameter decreases the FoM. It is observed that this behavior is consistent with the penetration depth results shown in Figure 12a, where an increase in chirality typically increases the penetration depth and consequently broadens the spatial extent of the modal field. The corresponding shift in the FoM curves toward higher frequencies also indicates that chirality alters the accessible propagation band. A different behavior is observed in Case II, as shown in Figure 13b. The FoM increases with increasing chirality, suggesting that the effect of chirality on the balance between propagation loss and transverse confinement is opposite to that observed in Case I. For Case II, the modification of the modal attenuation and field localization caused by chiral coupling results in a higher propagation-to-confinement ratio at higher chirality values. The opposite chirality dependence of the FoM in the two cases is particularly significant for device design. The two UAC configurations, therefore, provide distinct approaches to controlling the trade-off between SPP confinement and propagation loss. Although the present theoretical model does not directly determine the modulation bandwidth or insertion loss of a complete device, it provides useful first-order metrics for evaluating whether the device is suitable for integrated optical interconnects based on its propagation loss, penetration depth, and FoM calculations. In this work, the UAC layer is treated as an effective anisotropic chiral medium characterized by prescribed transverse and longitudinal permittivity components and a controllable chirality parameter. It is possible to achieve such effective constitutive responses using engineered chiral inclusions embedded within anisotropic host media [29]. To conduct an experimental study, an appropriate metamaterial or composite medium would need to be designed, followed by characterization of permittivity and chirality in the targeted near-infrared frequency range. The assumed UAC medium should therefore be interpreted as a theoretical design parameter, whereas experimental validation remains a key direction for future work [47,48].

## 4. Conclusions

In this paper, we examined the propagation characteristics of SPPs at the InSb-UAC-InSb interface in the near-infrared frequency regime. The electromagnetic field components were derived using Maxwell’s equations, and the dispersion relation was obtained by applying the appropriate boundary conditions. The propagation constant was plotted against incident-wave frequency and InSb temperature to evaluate the influence of material parameters. The propagation characteristics of the EM wave in the proposed geometry are found to be temperature-dependent. The influence of chirality, core-width, InSb temperature, and operating frequency on the propagation constant was analyzed. The numerical results show that these characteristics depend strongly on the physical parameters. Two different types of uniaxial chiral (UAC) media were investigated. Based on the numerical analysis, Case I waveguides are more suitable at higher frequencies, whereas Case II waveguides are effective at lower frequencies and provide enhanced field confinement and low propagation loss. The normalized electromagnetic field profiles confirm the surface-wave nature of the supported modes, while the penetration depth analysis quantifies their transverse spatial confinement. Furthermore, the FoM analysis in Cases I and II indicates distinct chirality-dependent trade-offs between propagation loss and confinement. The frequency- and temperature-based analysis opens new possibilities for tunable plasmonic devices in the near-infrared frequency regime. The present work provides additional freedom for designing and fabricating waveguides, thermally reconfigurable photonic components, sensors, and near-infrared optical communication devices.

## Data Availability

Details about the data are provided in the article.
